# Retraction: Challenges and opportunities of hydrothermal carbonisation in the UK; case study in Chirnside

**DOI:** 10.1039/d1ra90160e

**Published:** 2021-10-19

**Authors:** Eloise Bevan, Jile Fu, Ying Zheng

**Affiliations:** Institute for Materials and Processes, School of Engineering, The University of Edinburgh Edinburgh EH9 3FB UK ying.zheng@uwo.ca; Department of Chemical and Biochemical Engineering, Western University London Ontario N6A 5B9 Canada

## Abstract

Retraction of ‘Challenges and opportunities of hydrothermal carbonisation in the UK; case study in Chirnside’ by Eloise Bevan *et al.*, *RSC Adv.*, 2020, **10**, 31586–31610, DOI: 10.1039/d0ra04607h.

We, the authors, hereby wholly retract this article due to an error in the calculations. In this article, only the electrical energy demand of Chirnside’s residents was considered and the total energy supply from the hydrochar produced by the modelled plant was taken as the total HHV (higher heating value) energy content contained in these pellets. Thus, the following calculations did not consider the efficiencies in the conversion of hydrochar into its usable forms of energy (electricity and thermal energies). Therefore, the statement that HTC can provide Chirnside’s residents with 35.6% of their energy demand is fundamentally incorrect.

Having consulted with an independent expert, the Royal Society of Chemistry has determined that any changes made to the paper to correct this would be major, and therefore that the best course of action is retraction and republication of the article with the correct data. The Royal Society of Chemistry is happy that the overall conclusions of the paper are not affected by this error, and therefore that republication of the work with the correct data is appropriate. The republished article was peer reviewed and can be found at https://doi.org/10.1039/D1RA06736B.

We, the authors, brought this matter to the attention of the Royal Society of Chemistry ourselves, and are happy with the decision to retract and republish this article.

Signed: Eloise Bevan, Jile Fu and Ying Zheng

Date: 15/10/2021

Retraction endorsed by Laura Fisher, Executive Editor, *RSC Advances*

## Supplementary Material

